# A systematic review of neonatal treatment intensity scores and their potential application in low-resource setting hospitals for predicting mortality, morbidity and estimating resource use

**DOI:** 10.1186/s13643-017-0649-6

**Published:** 2017-12-07

**Authors:** Jalemba Aluvaala, Gary S. Collins, Michuki Maina, James A. Berkley, Mike English

**Affiliations:** 1 KEMRI-Wellcome Trust Research Programme, P.O Box 43640 – 00100, Nairobi, Kenya; 2 Department of Paediatrics and Child Health, College of Health Sciences, University of Nairobi, Kenyatta National Hospital, P. O. Box 19676-00202, Nairobi, Kenya; 3 Centre for Tropical Medicine and Global Health, Nuffield Department of Medicine, University of Oxford, Oxford, OX3 7FZ UK; 4 The Childhood Acute Illness & Nutrition (CHAIN) Network, P.O Box 43640 – 00100, Nairobi, Kenya; 5 Centre for Statistics in Medicine, Nuffield Department of Orthopaedics, Rheumatology and Musculoskeletal Sciences, Botnar Research Centre, University of Oxford, Oxford, OX3 7LD UK

**Keywords:** Neonatal prognosis, Treatment intensity, Prediction model, CHARMS

## Abstract

**Background:**

Treatment intensity scores can predict mortality and estimate resource use. They may therefore be of interest for essential neonatal care in low resource settings where neonatal mortality remains high. We sought to systematically review neonatal treatment intensity scores to (1) assess the level of evidence on predictive performance in predicting clinical outcomes and estimating resource utilisation and (2) assess the applicability of the identified models to decision making for neonatal care in low resource settings.

**Methods:**

We conducted a systematic search of PubMed, EMBASE (OVID), CINAHL, Global Health Library (Global index, WHO) and Google Scholar to identify studies published up until 21 December 2016. Included were all articles that used treatments as predictors in neonatal models. Individual studies were appraised using the CHecklist for critical Appraisal and data extraction for systematic Reviews of prediction Modelling Studies (CHARMS). In addition, Grading of Recommendations Assessment, Development, and Evaluation (GRADE) was used as a guiding framework to assess certainty in the evidence for predicting outcomes across studies.

**Results:**

Three thousand two hundred forty-nine articles were screened, of which ten articles were included in the review. All of the studies were conducted in neonatal intensive care units with sample sizes ranging from 22 to 9978, with a median of 163. Two articles reported model development, while eight reported external application of existing models to new populations. Meta-analysis was not possible due heterogeneity in the conduct and reporting of the identified studies. Discrimination as assessed by area under receiver operating characteristic curve was reported for in-hospital mortality, median 0.84 (range 0.75–0.96, three studies), early adverse outcome and late adverse outcome (0.78 and 0.59, respectively, one study).

**Conclusion:**

Existing neonatal treatment intensity models show promise in predicting mortality and morbidity. There is however low certainty in the evidence on their performance in essential neonatal care in low resource settings as all studies had methodological limitations and were conducted in intensive care. The approach may however be developed further for low resource settings like Kenya because treatment data may be easier to obtain compared to measures of physiological status.

**Systematic review registration:**

PROSPERO CRD42016034205

**Electronic supplementary material:**

The online version of this article (10.1186/s13643-017-0649-6) contains supplementary material, which is available to authorized users.

## Background

Improving neonatal care is now a global concern, and tools to examine system performance and guide service planning in low and middle income countries (LMIC) are needed. Higher quality of care across a broader range of settings is necessary if LMICs are to realise the substantial reduction in neonatal mortality expected from the delivery of essential interventions at scale in facilities [1, 2]. Clinical prediction models are typically used to support shared decision making at individual patient level, for risk stratification in clinical trials or for case-mix adjustment in quality of care assessments [3, 4]. They have also been developed to estimate resource use and thereby inform service delivery planning [3, 5]. By facilitating better decision making, prediction models could contribute to the improvement of the quality of hospital care for neonates in LMICs ultimately improving neonatal survival.

In considering the use of prediction models to support decision making in hospital-based essential neonatal care in LMICs, we may either develop a new model or choose from amongst existing models. Collins and colleagues recommend the latter approach before developing new models to avoid waste of resources [6, 7]. Selecting from amongst existing models should however be guided by the evaluation of existing models for performance and suitability to the context [8]. Prediction models have variably been termed as prediction rules, probability assessments, prediction models, decision rules and risk scores [9]. Existing models that predict in-hospital mortality were identified from published reviews of neonatal models and summarised in Additional file 1 [10–18]. This overview revealed that the neonatal therapeutic intervention scoring system (NTISS) is unique amongst neonatal prediction models as it uses treatments rather than clinical and pathophysiological factors as predictors [10]. The NTISS predicts in-hospital mortality and morbidity in addition to estimating resource utilisation, particularly nursing workload [19]. The latter may help identify service delivery bottlenecks in essential neonatal care providing information to guide strategic planning [20].

Existing neonatal prediction models have typically been developed for settings offering advanced neonatal intensive care including mechanical ventilation and other expensive interventions. These are not directly applicable to settings offering only essential neonatal care where respiratory support is limited to oxygen via nasal cannula without monitoring such as pulse oximetry [21, 22]. Amongst the models included in the published reviews, only one was developed specifically for a low-resource setting, the simplified age-weight-sex (SAWS) [23]. This was, however, not considered further as it was developed for very low birth weight neonates only. We therefore conducted a systematic review to systematically identify and characterise prediction model research that has used treatments as predictors (treatment intensity models) in neonatal care specifically to (1) assess the certainty of evidence on predictive performance in predicting clinical outcomes (primarily in-hospital mortality) and estimating resource utilisation and (2) assess the applicability of the identified models to neonatal care in LMIC.

## Methods

The systematic review was conducted following the recently published guidance from the Cochrane Prognosis Methods group; the CHecklist for critical Appraisal and data extraction for systematic Reviews of prediction Modelling Studies (CHARMS) [24]. In addition, the Grading of Recommendations Assessment, Development, and Evaluation (GRADE) was used as a guiding framework to assess the quality of the retrieved articles [25]. Reporting of the review was done using the Preferred Reporting Items for Systematic Reviews and Meta-Analyses (PRISMA) recommendations (Additional file 2) [26]. Details of the protocol for this systematic review were registered on the international prospective register of systematic reviews (PROSPERO): Jalemba Aluvaala, Gary Collins, Michuki Maina, James Berkley, Mike English. Neonatal treatment intensity scores and their potential application for low resource settings: a systematic review. PROSPERO 2016: CRD42016034205. Available from: http://www.crd.york.ac.uk/PROSPERO/display_record.asp?ID=CRD42016034205.

Registration of the protocol was done after the initial screening of articles.

### Search strategy

The primary electronic database used was PubMed with supplementary searches conducted in EMBASE (OVID), CINAHL, Global Health Library (Global index, WHO) and Google Scholar. The last search conducted was on 21 December 2016. Bibliographies of identified papers were also hand-searched for additional papers.

The following key search terms were used “neonate” or “neonatal or newborn”, “treatment or therapeutic” or “therapy”, “intensity” and “score or scoring”. In PubMed, the search strategy was implemented using medical subject headlines (MeSH) where applicable and the appropriate Boolean terms, ((("Neonatology"[Mesh] OR "Infant, Newborn"[Mesh]) AND ("Therapeutics"[Mesh] OR "therapy"[Subheading])) AND intensity) AND scor*. A similar approach was used in all the other electronic databases. The search strategy was developed with input from an expert medical librarian. No language restrictions were applied on the selection of the articles.

The primary search strategy was augmented by substituting a validated search string for prediction models in PubMed for the scor* term used in the first search string; ((Validat* OR Predict* OR Rule*) OR (Predict* AND (Outcome* OR Risk* OR Model*)) OR ((History OR Variable* OR Criteria OR Scor* OR Characteristic* OR Finding* OR Factor*) AND (Predict* OR Model* OR Decision* OR Identif* OR Prognos*)) OR (Decision* AND (Model* OR Clinical* OR Logistic Models/)) OR (Prognostic AND (History OR Variable* OR Criteria OR Scor* OR Characteristic* OR Finding* OR Factor* OR Model*))) [27].

### Screening process

All articles identified by the search were initially screened for eligibility on title independently by two reviewers (JA, ME) with disagreements resolved by discussion. The search results were exported to the reference management software EndNote X7 (Thomas Reuters, Philadelphia, USA). Duplicate articles were removed and the remaining titles and abstracts screened. Full-text articles were retrieved and assessed for eligibility using predefined criteria (Table 1) for inclusion in the review. The target population was neonates defined as babies aged 0–28 days (Table 1).

**Table 1:** Eligibility criteria for inclusion in the review

Criteria	Inclusion	Exclusion
Model type	Prognostic models	Diagnostic models*
Intended scope of the review	Inform decision making at individual level (e.g. using risk of in-hospital mortality) and planning service delivery (e.g. nursing staffing)	
Types of modelling studies	Development and/or validation	
Target population	Neonates† admitted to a neonatal unit in any country	Studies limited to neonates with congenital anomalies older children or adults
Predictors	Any use of treatments or interventions as predictors	Non-therapeutic intervention, e.g. radiological imaging intensity Treatment intensity not measured by enumeration of therapeutic interventions
Outcomes	Any outcome	
Time of prediction	No restriction	
Intended moment of use	No restriction	

*Diagnostic models estimate probability that a particular disease is currently present in an individual in contrast to prognostic models that estimate probability of future events

†Neonate defined as a baby aged 0–28 days and all the articles included adhered to this definition

### Data extraction and critical appraisal of individual studies

Two independent reviewers (JA, MM) extracted data using a standardised form based on the CHARMS checklist, and any disagreements were resolved by discussion. Data elements extracted were study design, participants, geographical location, outcomes predicted, description of model development (type of model, e.g. logistic regression), number and type of predictors, number of study participants and number of outcome events, handling of missing data and model performance (calibration, discrimination). Critical appraisal of individual studies was by applying the CHARMS guidance on each of the elements of data extracted to assess potential limitations [24].

### Descriptive analyses

A quantitative meta-analysis was not conducted due to heterogeneity in the conduct and reporting of the identified studies. Results were therefore summarised descriptively and synthesised using a narrative approach.

### Certainty of the evidence across studies

In the absence of a specific tool for assessing risk of bias across studies for clinical prediction models, the GRADE approach was used as a guiding framework for this purpose [28]. GRADE assesses the certainty of the evidence in the estimates of effects for given outcomes across studies. In general, this is achieved using explicit criteria including study design, risk of bias, imprecision, inconsistency, indirectness and magnitude of effect [28]. For this review, the GRADE approach for diagnostic studies was used as a guide (Additional file 3) [29–31]. Certainty in the evidence for each outcome across the identified articles was initially rated as high quality if the studies were prospective cohort as recommended by CHARMS [24]. Subsequently, certainty was downgraded if there were serious limitations in the conduct of the studies as defined by CHARMS and if there was inconsistency, indirectness, imprecision and publication bias as defined by GRADE (Additional file 4). Certainty of the evidence on predictive performance was thus rated as high, moderate, low or very low for each outcome.

## Results

### Study selection

**Fig. 1:**
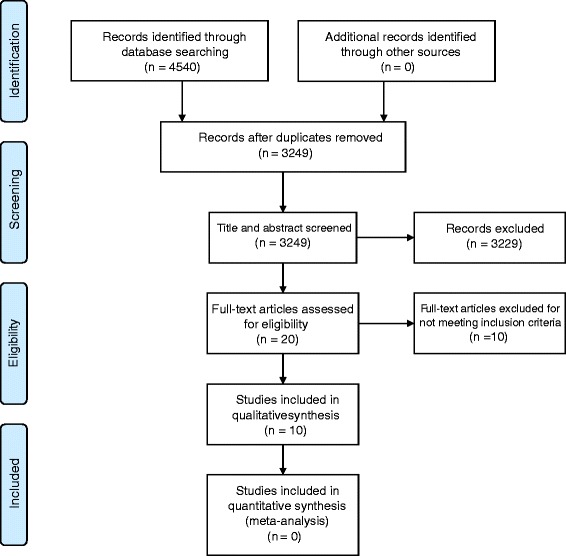
Flow diagram for the selection of studies on neonatal treatment intensity models

A total of 3249 unique articles were identified by the search strategy, of which 3229 were excluded based on the title and abstract. The full texts of 20 articles were screened, of which 10 articles met the inclusion criteria and were included in this review (Fig. 1). Articles were excluded for the following reasons: a population other than neonates was studied, intensity referred to a non-therapeutic intervention (e.g. radiological imaging intensity), treatment intensity was measured by means other than enumeration of therapeutic interventions provided (e.g. proportion of days that hospital intensive care was required) and studies limited to neonates with congenital anomalies considered to be lethal.

### Study characteristics

Table 2 provides information on the general characteristics of the included studies. All of the studies were conducted in tertiary neonatal intensive care units (NICU) with five single centre and five multicentre studies and included 12,899 neonates. Sample sizes ranged from 22 to 9978 with a median of 163. Two out of the ten articles reported model development [19, 32]. Eight were reports of external application of existing models to new populations. Gray and colleagues developed the NTISS while Shah et al. developed a score using selected variables derived from the NTISS [19, 32]. Seven of the articles reporting external application to new populations used the NTISS [33–39]. One study used a therapeutic score originally developed for adults, the therapeutic intervention scoring system (TISS) [40].

**Table 2:** Characteristics of studies included in the review

Study	Study dates	Objective*	Setting	Sample size	In-hospital mortality^||^
Georgieff, 1989 [40]	1987	Validation (TISS)^‡^	1 NICU†, USA	55	0
Gray, 1992 [19]	1989–1990	Development (NTISS)^§^	3 NICUs†, USA	1768	114
Davies, 1995 [33]	Not reported	Validation (NTISS)^§^	1 NICU†, South Africa	50	8
Eriksson, 2002 [34]	1991–1995	Validation (NTISS)^§^	2 NICUs†, Sweden	240	39
Zupancic, 2002 [35]	1998 & 1999	Validation (NTISS)^§^	1 NICU†, USA	154	Not reported
Mendes, 2006 [35]	2004	Validation (NTISS)^§^	2 NICUs†, Brazil	96	9
Rojas, 2011 [37]	2007	Validation (NTISS)^§^	1 NICU†, 1 intermediate unit, Colombia	22	Not reported
Oygur, 2012 [38]	2006–2010	Validation (NTISS)^§^	1 NICU†, Turkey	364	103
Shah, 2015 [32]	2010–2012	Development (unnamed)	23 NICU†s, Canada	9978	650
Wu,2015 [39]	2007–2011	Validation (NTISS)^§^	1 NICU†, Taiwan	172	18

*Study objective, model development (creation of a new model) or validation (application of an existing score/model to an external population)

†Neonatal intensive care unit

‡Therapeutic intervention scoring system

§Neonatal therapeutic intervention scoring system

||Primary outcome for the review. Data was however extracted on all outcomes reported by the authors

### Critical appraisal of study design and statistical analysis of individual studies

#### All studies

All of the included studies were individually critically appraised based on the limitations in the design of the study (Table 3) and limitations in the statistical analysis (Table 4). With regard to study design, four were prospective studies, two combined prospective and retrospective data, while four were retrospective (Table 3). With regard to the selection of participants, the majority of the studies (8/10) recruited all eligible infants and therefore were deemed to have no selection bias. None of the articles explicitly reported blinding during collection of data on predictors and outcomes (Table 3). However, this does not constitute a risk of bias for mortality in all instances and where a prospective study design was used for all outcomes. Missing data may also give rise to bias during analysis, and only one study was judged to have no risk of bias due to missingness [32].

**Table 3:** Limitations in individual studies with respect to study design and data collection

Study	Study type*	Participants^†^	Outcome(s)^‡^	Predictors^§^	Sample size^||^	Missing data^¶^
Georgieff,1989 [40]	-	-	+	+	+	?
Gray,1992 [19]	-	-	+	+	?	+
Davies,1995 [33]	+	+	+	+	+	?
Eriksson,2002 [34]	-	-	+	+	+	+
Zupancic,2002 [35]	-	+	?	+	+	?
Mendes,2006 [36]	-	-	+	+	+	+
Rojas,2011 [37]	-	-	+	+	+	?
Oygur,2012 [38]	+	-	+	+	+	-
Shah,2015 [32]	+	-	+	+	?	+
Wu,2015 [39]	+	-	+	+	+	?

+ Limitation present

- No limitation

? Not reported therefore unclear risk of bias

Limitation present if (based on CHARMS criteria):

* Data collection not prospective

† Not all eligible neonates recruited resulting in risk of selection bias

‡ Risk of measurement error in determining outcome status

§ Risk of measurement error in determining predictor status

|| Sample size less than the recommended

¶ Missing data causing risk of bias

**Table 4:** Limitations in individual studies with respect to statistical analysis

Study	Study type	Performance^†^	Validation^‡^	Presentation^§^
Georgieff,1989 [40]	Evaluation of existing score*	?	?	?
Gray, 1992 [19]	Development of new score	+	?	?
Davies, 1995 [33]	Evaluation of existing score*	?	?	?
Eriksson, 2002 [34]	Evaluation of existing score*	+	?	?
Zupancic, 2002 [35]	Evaluation of existing score*	?	?	?
Mendes, 2006 [36]	Evaluation of existing score*	?	?	?
Rojas, 2011 [37]	Evaluation of existing score*	?	?	?
Oygur,2012 [38]	Evaluation of existing score*	+	?	?
Shah et al., 2015 [32]	Development of new score	+	?	?
Wu, 2015 [39]	Evaluation of existing score*	+	?	?

**+** Limitation present (based on CHARMS criteria)

**-** No limitation

? Not reported

* None of these applied a regression formula from the original score development study to the new population but instead specified new models thus were model re-development rather than external validation studies

† Limitation present if (based on CHARMS criteria) either score discrimination OR calibration only was reported

‡ Internal validation (to quantify model overfitting) OR external validation (model performance in new population)

§ Presentation of final model as either a regression formula or a score chart

#### Score development studies

Two studies described the development of a treatment intensity score [19, 32]. The NTISS was developed to serve as a “therapy-based severity-of-illness tool for use in intensive care” by modification of the adult TISS [19, 41]. Included in the NTISS were 63 therapeutic interventions delivered in neonatal intensive care, e.g. surfactant and mechanical ventilation [19]. Shah and colleagues on the other hand used 24 NICU therapeutic interventions (many that are included in the NTISS) to develop a score to measure intensity of NICU resource use [32]. In both of these studies, treatment predictor selection and their respective predictor weights were determined by consensus amongst experts. With respect to prediction of mortality, there were a total number of 114 deaths (events) in Gray et al. and 650 deaths in Shah et al. (Table 3) [19, 32]. From a predictive modelling perspective, it is recommended that there should be at least ten outcome events (deaths in this case) for each predictor variable in the regression model [24]. It was however not possible to assess for risk of overfitting on the basis of the sample size since none of the two studies published the final regression models (Table 4), and therefore, it is not clear what the number of events per predictor (EPV) were.

Neither of the two score development studies specified a regression model that included the therapeutic interventions as individual predictors. Rather, the therapeutic interventions were assigned sub-scores by an expert panel which were then summated to give a total score for each patient. The statistical relationship between the total scores and the outcomes was then examined. Nonetheless, Shah et al. reported model discrimination with a c-statistic of a regression model (Table 4) [32]. The study describing the development of the NTISS did not report model discrimination but assessed calibration using the Hosmer-Lemeshow (H-L) technique (Table 4) [19]. Finally, neither of these two studies presented a final model or score chart to demonstrate how to determine predicted probabilities of the outcomes for individual patients.

#### Application of existing scores to external populations

Eight studies described the evaluation of existing therapeutic intensity models in a different geographical setting but still in neonatal intensive care. Seven studies evaluated the NTISS [33–39]. A single study evaluated a version of the adult TISS in neonates [40]. For external validation studies using regression approaches, recommended sample sizes of between 100 and 250 outcome events have been suggested [42–44]. With respect to the primary outcome, (in-hospital mortality) only three studies had at least 100 outcome events (Table 2) [32, 38, 45].

Zupancic et al. used individual treatments as separate parameters in a linear regression model with personnel time as outcome [35]. Oygur et al. also had individual treatments as separate parameters but did not specify the type of model used to predict in-hospital mortality [38]. In contrast, the other six studies summated the treatments to give a single score which was then used in subsequent analyses as a single parameter [33, 34, 36, 37, 39, 40]. This is the same approach used by Gray et al. in developing the NTISS [19]. In terms of model performance, Eriksson et al., Oygur et al. and Wu et al. computed discrimination using area under the receiver operating characteristic curve (AUROC) but did not report model calibration [34, 38, 39]. None of these eight studies applied the exact model formula as obtained from the original score development work but instead conducted fresh analyses; these were therefore all in effect model re-development studies in a new population rather than external validation [24, 46, 47].

### Synthesis of results of estimated performance across studies

The primary outcomes of interest were mortality and nursing workload. However, for the narrative synthesis of the results, we determined that outcomes were reported in three main categories: mortality, morbidity and resource utilisation (including nursing workload) across the ten articles. There are two distinct aspects of performance for statistical models, predictive (measures include discrimination and calibration) or explanatory (testing causal relationships) [48, 49]. However, the two approaches are often conflated creating ambiguity, and this distinction is therefore made in the narrative synthesis [49].

#### Performance with respect to mortality

Five studies reported on in-hospital mortality as an outcome (Table 5, Additional file 5). No other mortality outcome measure was reported. Three of these computed discriminatory performance using AUROC analysis [34, 38, 39]. Calibration was reported in only one article [19]. Explanatory rather than predictive performance for in-hospital mortality was reported in three articles [19, 33, 39].

**Table 5:** Summary of certainty of evidence in predicting outcome and resource use using GRADE

Outcome	No. of studies	Factors that may decrease certainty of evidence*					Overall† certainty	Importance ‡
		Limitations	Indirectness	Inconsistency	Imprecision	Reporting bias		
Mortality	5 studies (*n* = 2594)	Serious	Serious	None	None	Unlikely	⊕⊕OO low	Critical
Morbidity	2 studies (*n* = 295)	Serious	Serious	None	None	Unlikely	⊕⊕OO low	Critical
Composite (Morbidity and Mortality)	2 studies (*n* = 10,218)	Serious	Serious	None	None	Unlikely	⊕⊕OO low	Important
Nursing workload	3 studies (*n* = 317)	Serious	Serious	None	None	Unlikely	⊕⊕OO low	Critical
Hospital Costs	1 study (*n* = 1768)	Serious	Serious	None	None	Unlikely	⊕⊕OO low	Important
Length of stay	1 study (*n* = 1768)	Serious	Serious	None	None	Unlikely	⊕⊕OO low	Important
Time inputs	1 study (*n* = 154)	Serious	Serious	None	None	Unlikely	⊕⊕OO low	Critical
Comparison of resource use	1 study (*n* = 96)	Serious	Serious	None	None	Unlikely	⊕⊕OO low	Important
Rehabilitation	1 study (*n* = 240)	Serious	Serious	None	None	Unlikely	⊕⊕OO low	Not important

* From GRADE, Grading of Recommendations Applicability, Development and Evaluation

†Certainty rating scale; high (⊕⊕⊕⊕), moderate, low, very low (OOOO)

‡Importance of outcomes (from GRADE)

In Eriksson et al., AUROC for in-hospital mortality (39 deaths) was 0.82(SE 0.04) [34]. Oygur et al. reported performance with 63 treatment variables and with sensitive treatments only (based on significant association with in-hospital mortality by chi-square test). The AUROC for all treatment variables (63 treatments) by birth weight categories were 500–1499 g (103 deaths), 0.851 (95% CI 0.809–0.885);1000–1499 g (33 deaths), 0.834 (95% CI 0.781–0.878); and 500–999 g (70 deaths), 0.749 (95%CI 0.662–0.822). Using Student’s *t* test to compare AUROCs for all variables (63) versus sensitive variables only (18 treatments) resulted in a significant change for the 500–999 g (70 deaths) category only, 0.749 vs 0.823(*P* = 0.02) [38].

Performance of NTISS 24 h after admission was examined by Wu et al. who compared serial scores in preterm infants weighing < 1500 g (18 deaths) and found AUROC at 24 h, 0.913; 48 h, 0.955; and 72 h, 0. 958. Confidence intervals for the AUROC were not provided, but using Tukey’s honest significant difference (HSD) test, there was significant difference between the 24 h average score when compared to both 48 and 72 h average scores (*p* < 0.001). There was no difference between the 48 and 72 h scores (*p* = 0.391) [39].

With regard to explanatory performance, Gray et al. found that NTISS correlated with in-hospital mortality [19]. Davies et al. found no difference between predicted mortality with actual mortality [33], while Wu and colleagues found that average NTISS scores at 24, 48, and 72 after admission were higher in the mortality group than in those who survived [39].

#### Performance with respect to morbidity

Predictive performance for morbidity was reported by Eriksson et al. only; here NTISS poorly predicted morbidity at 4 years of age (growth retardation, neurodevelopmental impairment, pulmonary problems) with AUROC of 0.59 (SE 0.05) but calibration was not reported (Table 5, Additional file 5) [34]. Explanatory performance was reported by Georgieff and colleagues who found a linear relationship between severity of physiologic instability and the TISS. In addition, the mean TISS was significantly higher in infants discharged after 14 days and those with severe lung disease (hyaline membrane disease (HMD)) [40].

#### Performance with respect to composite of mortality and morbidity

Predictive performance for the composite outcome of morbidity and mortality was reported in two articles (Table 5, Additional file 5). In the first, Eriksson et al. found that for early adverse outcome (all-cause mortality or oxygen dependence, intraventricular haemorrhage/periventricular leukomalacia or retinopathy of prematurity), the AUROC curve for NTISS was 0.78 (SE 0.03) [34]. Shah et al. reported a c-statistic of 0.86 for a composite outcome (all-cause mortality, ≥ grade 3 IVH, PVL, stage ≥ 3 ROP, oxygen dependence or stage ≥ necrotizing enterocolitis) from a logistic regression model that included therapeutic intensity, NICU size, NICU occupancy rate, gestational age, small for gestational age, multiple births, outborn, caesarean delivery, SNAP II score > 20 and mechanical ventilation).

#### Performance with respect to estimating resource utilisation

Four studies estimated the extent to which resource use (measured in different ways) was explained by treatment intensity (Table 5, Additional file 5). There was statistical association between nursing workload and both TISS and NTISS [19, 35, 37, 40]. In addition, the NTISS correlated with hospital costs and length of stay [19].

### Critical appraisal of certainty of the evidence in model performance across all studies

GRADE criteria (Additional file 3 and Additional file 4) were used to assess the certainty in the evidence of predictive performance for each outcome *across all* studies where it was reported and tabulated (Table 5). The outcomes were reported in three main categories: mortality, morbidity and resource utilisation (nursing workload, length of stay, time inputs, comparison of resource use). Table 5 shows that there is low certainty in the evidence of predictive performance for these three categories of outcomes for low-resource settings as all the studies had serious limitations in their conduct (as determined by CHARMS) in addition to indirectness as they were all conducted in high-resource settings (GRADE).

## Discussion

### Performance of neonatal treatment intensity models in model development studies

Existing neonatal treatment intensity models can predict mortality and morbidity. Discriminatory performance as measured by the area under the receiver operating curve is poorest for long-term morbidity (0.59) and highest for in-hospital mortality in infants weighing 1000–1499 g for NTISS measured at 72 h post admission (0.958) [34, 39]. Calibration was reported by Gray et al. only who found a close agreement between observed and predicted in-hospital mortality by the Hosmer-Lemeshow test [19]. Using a variety of tests of statistical association rather than predictive performance, treatment intensity was found also to be associated with mortality, morbidity and resource utilisation [19, 35, 37, 39, 40].

Discriminatory performance of neonatal treatment intensity models for predicting in-hospital mortality measured by AUROC ranges from 0.749 to 0.958 [38, 39]. The recommended threshold for good discrimination is 0.8 [11]. The reported performance therefore suggests that the treatment scoring approach may perform well in distinguishing between neonates who die and those who do not in neonatal intensive care. This discriminatory ability is comparable to that of more commonly used neonatal predictive models. Discriminatory performance at model development in the prediction of in-hospital mortality measured by AUROC ranges from 0.87 (SE 0.33) for the Transport Risk Index for Physiological Stability version 2; TRIPS-II [50] to 0.92 (SE 0.01) for the Clinical Risk Index for Babies version 2; CRIB II [51]. Despite the difference in predictors, there is thus consistency in the evidence that clinical prediction modelling approaches may be useful in predicting in-hospital mortality in neonatal medicine. The application of the treatment intensity scoring approach in adult and paediatric intensive care preceded use in the neonatal population. However, none of these adult or paediatric studies reported the discriminatory performance of the scores [41, 52–55].

With regard to calibration, Gray et al. reported good calibration in predicting for in-hospital mortality by the NTISS using the H-L test. This means that the predicted deaths closely matched the observed deaths. Similarly, good calibration in predicting in-hospital mortality as measured by the H-L statistic has been reported for the physiologic scores Score for Neonatal Acute Physiology – Perinatal Extension version II (SNAP-PE II) [56], CRIB II [51] and TRIPS II [50]. These results must however be interpreted with caution given the limitations of the H-L test [57, 58]. None of the included studies reported calibration using the preferred method, calibration plots [57].

There is however low certainty in this evidence of performance with reference to essential neonatal care in low resource settings for two reasons. One, there were serious limitations identified in the design and conduct of these studies such that even for high-income countries, the certainty in score performance would be low as well. Secondly, these results are not directly generalisable to essential neonatal care in low-resource settings as the studies were all conducted in neonatal intensive care in high-income settings given the differences in range of treatments, staffing and case-mix.

Whereas the focus of this work was to investigate predictive performance of these scores, the retrieved articles also reported on explanatory performance. Treatment intensity was found to be associated with mortality, morbidity and resource utilisation [19, 35, 37, 39, 40]. This is consistent with findings in adult and paediatric intensive care for mortality [41, 54]. Similarly, treatment intensity was also found to be associated with nursing workload and could be used to determine the workload a typical nurse is capable of per shift in adult intensive care [41, 55]. Finally, treatment intensity also correlates with costs in adult intensive care [41]. The frequent reporting of such analyses alongside predictive analyses underscores the importance of clearly distinguishing predictive from explanatory aspects of models [49, 59].

### Generalisability to essential neonatal care in low-resource settings

The second objective of this review was concerned with generalisability of the identified treatment intensity scores to essential neonatal care in low-resource settings. Generalisability is assessed by evaluating the performance of previously developed models in a new population, i.e. external validation [58]. There were no studies of the treatment intensity models in low-resource settings identified. The eight studies which applied previously developed treatment intensity models to new populations were potentially external validation studies [33–40]. However, none of these applied model coefficients derived from the derivation studies to the new populations to compute the predicted probabilities of the outcomes to allow comparison with the observed outcomes. None of the studies therefore qualified as an external validation.

The use of treatments as predictors presents three potential limitations. To begin with, the treatments prescribed are dependent on the availability of resources and may not necessarily be an accurate reflection of the patient’s actual requirements. Secondly, there may be a variation in clinical practice which will be reflected in differences in treatments and thus potentially in the model performance. Thirdly, the treatment predictors used as predictors in the identified studies reflect the neonatal intensive care study sites where mechanical ventilation and exogenous surfactant amongst other expensive and invasive interventions are available. These cannot be applied directly to populations receiving essential neonatal care in low-resource settings in LMICs, for example with respiratory support limited to oxygen via nasal cannula. However, the treatment intensity approach has potential in the Kenyan context since there is a relatively high degree of standardisation of essential neonatal care in the first referral level facilities which mitigates these limitations to some extent [60].

### Limitations of the review

The systematic review was guided by the CHARMS recommendations which provide a clear guide on extracting data and identifying limitations in individual studies. However, it does not provide guidance on how to assess the quality of evidence on individual outcomes across all the identified studies. As a result, the GRADE approach was used as a guiding framework for this purpose. While there is no GRADE guideline specifically for clinical prediction models, GRADE for diagnostic models was judged sufficiently similar to and used for this review. A key strength of the GRADE approach is that the judgements on the quality of evidence are made on explicit criteria. Nonetheless, the PROBAST (prediction study risk of bias assessment tool) tool which is specific for this purpose is under development [61].

## Conclusion

Existing neonatal treatment intensity scores show promise in predicting mortality and morbidity, but there is low certainty in the evidence on their performance in essential neonatal care in low resource settings in LMICs. The limitations in the included studies mirror those reported in other systematic reviews including unclear study designs, failure to report follow-up times, sample size calculations and performance measures [5, 62–66]. However, over 12,000 patients were included in this review of ten studies compared to 467 patients in the only model developed to date for neonates in low-income countries (SAW) [23]. In addition, increase in treatment intensity is associated with higher nursing workload, hospital costs and length of stay. The approach may therefore be usefully developed further for low-resource settings, e.g. Kenya because treatment data may be easier to obtain compared to other parameters like measures of physiological status [67]. This will entail addressing the limitations identified by applying appropriate methods for model development, validation and most importantly the evaluation of clinical utility and impact [48, 57, 68–70]. Prognostic information obtained in this way can support decision making in the planning and organisation of quality essential neonatal care.

## Additional files

Additional file 1: Summary of prognostic models for predicting in-hospital neonatal mortality. Comparison of predictors and outcomes of neonatal prognostic models included in published reviews. (DOCX 15 kb)

Additional file 2: PRISMA checklist. Completed PRISMA checklist. (DOC 63 kb)

Additional file 3: Application of GRADE as a guide in assessing certainty of evidence in prediction modelling studies. GRADE* criteria that were used as a guiding framework in assessing the certainty in the evidence for outcomes across the identified studies. *Grading of Recommendations Assessment, Development and Evaluation. (DOCX 14 kb)

Additional file 4: Using GRADE* as a guiding framework in rating of certainty of evidence in predictive model predictive performance. Description of the four categories in GRADE for rating certainty of evidence. *Grading of Recommendations Assessment, Development and Evaluation. (DOCX 13 kb)

Additional file 5: Summary of results from individual studies. Summary of data extracted from each eligible article. (DOCX 17 kb)

## Abbreviations

AUROC: Area under the receiver operating characteristic curve
CHARMS: CHecklist for critical Appraisal and data extraction for systematic Reviews of prediction Modelling Studies
CRIB: Clinical risk index for babies
GRADE: Grading of Recommendations Assessment, Development, and Evaluation
H-L: Hosmer-Lemeshow
HMD: Hyaline membrane disease
IVH: Intraventricular haemorrhage
LMIC: Low and middle income countries
NICU: Neonatal intensive care unit
NTISS: Neonatal therapeutic intervention scoring system
NUMIS: Nursing utilisation management information system
PRISMA: Preferred Reporting Items for Systematic Reviews and Meta-Analyses
PROBAST: Prediction study risk of bias assessment tool
PROSPERO: International prospective register of systematic reviews
PVL: Periventricular leukomalacia
ROP: Retinopathy of prematurity
SAW: Sex age weight score
SNAP: Score for neonatal acute physiology
SNAP-PE: Score for neonatal acute physiology – perinatal extension
TISS: Therapeutic intervention scoring system
TRIPS: Transport Risk Index for Physiological Stability
TTN: Transient tachypnoea of the newborn
