# Supplementary material for: A systematic review of neonatal treatment intensity scores and their potential application in low-resource setting hospitals for predicting mortality, morbidity and estimating resource use
Source: Syst Rev. 2017 Dec 7;6:248. doi: 10.1186/s13643-017-0649-6 (PMC5719732; doi:10.1186/s13643-017-0649-6)
Supplement: Supplementary file 1 — Summary of prognostic models for predicting in-hospital neonatal mortality. Comparison of predictors and outcomes of neonatal prognostic models included in published reviews. (DOCX 15 kb) [file 13643_2017_649_MOESM1_ESM.docx]

**Additional file 1: Table S1**. Summary of prognostic models for predicting in-hospital neonatal mortality

| Model | Model parameters | | Predicted outcomes | | |
| --- | --- | --- | --- | --- | --- |
|  | **Physiological** | **Therapeutic** | **In-hospital mortality** | **Morbidity** | **Resource use** |
| NTISS |  | 63 treatments and procedures in the first 24 hours e.g. oxygen, surfactant, antibiotics, phlebotomy | 🗸 | Length of stay | Costs, nursing workload |
| SNAP | 28 physiological parameters e.g. respiratory rate, heart rate, serum electrolytes |  | 🗸 | Correlation with NTISS |  |
| SNAP-II | Blood pressure, temperature, PO_2_/FIO_2,_ pH, seizures, urine output |  | 🗸 |  |  |
| SNAP-PE | SNAP plus birthweight, 5 min Apgar <7, Small for gestational age |  | 🗸 |  |  |
| SNAP-PE II | SNAP II plus birthweight ≤749g, 5 min Apgar <7, Small for gestational age, |  | 🗸 |  |  |
| NMPI | Gestational age, birthweight, cardiac arrest, PaO_2_/FIO_2,_ major malformations, sepsis, base excess |  | 🗸 |  |  |
| Sinkin | Birthweight, gestational age, 5 min Apgar, peak inspiratory pressure at 12 hours |  |  | Bronchopulmonary dysplasia |  |
| NBRS | pH, hypoglycaemia, intraventricular haemorrhage, periventricular leukomalacia, seizures, infection, need for mech. ventilation |  |  | Neurodisability |  |
| TRIPS | Temperature, respiratory status, blood pressure, response to noxious stimuli |  | 🗸 |  |  |
| TRIPS-II | Temperature, respiratory  status, blood pressure, response to noxious stimuli |  | 🗸 |  |  |
| VON-RA | Gestational age, gestational age squared, multiple gestation, outborn, Apgar score, gender, caesarean section, malformations |  | 🗸 |  |  |
| NQI-2* |  |  | 🗸 |  |  |

*NTISS* (Neonatal Therapeutic Intervention Scoring system), *SNAP* (Score for Neonatal Acute Physiology),

*SNAP-PE* (Score for Neonatal Acute Physiology-Perinatal Extension), *NMPI* (Neonatal Mortality Prognosis Index), *NBRS* (Nursery Neurobiologic Risk Score), *TRIPS* (Transport Risk Index of Physiologic Stability), *VON-RA* (Vermont Oxford Network Risk Adjustment), *NQI-2* (Neonatal Quality Indicator)

*Withdrawn, no longer available online
