# Supplementary material for: A systematic review of neonatal treatment intensity scores and their potential application in low-resource setting hospitals for predicting mortality, morbidity and estimating resource use
Source: Syst Rev. 2017 Dec 7;6:248. doi: 10.1186/s13643-017-0649-6 (PMC5719732; doi:10.1186/s13643-017-0649-6)
Supplement: Supplementary file 3 — Application of GRADE as a guide in assessing certainty of evidence in prediction modelling studies. GRADE* criteria that were used as a guiding framework in assessing the certainty in the evidence for outcomes across the identified studies. *Grading of Recommendations Assessment, Development and Evaluation. (DOCX 14 kb) [file 13643_2017_649_MOESM3_ESM.docx]

**Additional file 3: Table S2.** Application of GRADE as a guide in assessing certainty of evidence in prediction modelling studies**.**

| **Category** | **GRADE for Prediction Modelling Studies** |
| --- | --- |
| **1.Question formulation** | PICOT formulation; Population Issue Context Outcome Time  Formulate using CHARMS* guidance |
| **2.Outcomes** | Patient important outcomes, CHARMS guidance |
| **3.Rating the quality of evidence** | By outcome, across studies |
| **4.GRADE criteria for downgrading** |  |
| Risk of bias (RoB) | 1. First, assess RoB for each study. Use RoB domains in CHARMS* 2. Downgrading is based on considered judgment on the extent of bias in all studies reporting the outcome |
| Indirectness | Downgrading based on applicability issues. Use applicability domains of CHARMS* tool. |
| Inconsistency | Downgrading based on evaluation of unexplained heterogeneity.  Criteria are less clear compared to intervention studies, but include judgment on similarity of point estimates and extent of overlap of confidence intervals. Lack of appropriate statistical methods for assessing study heterogeneity. |
| Imprecision | Downgrading is based on evaluation of 95% confidence intervals around model discrimination and calibration. Formal guidance and methods on how this can be assessed not available yet. |
| Publication bias | Judgment of this criterion is challenging.  Existing methods used in intervention studies not applicable to prediction modelling studies |
| **5.GRADE criteria for upgrading** | Specific examples of situations in which upgrading is appropriate not yet available. |

* **CH**ecklist for critical **A**ppraisal and data extraction for systematic **R**eviews of prediction **M**odelling **S**tudies

Note: Adapted from Gopalakrishna G, Mustafa RA, Davenport C Et al. Applying Grading of Recommendations Assessment, Development and Evaluation (GRADE) to diagnostic tests was challenging but doable. Journal of Clinical Epidemiology. 2014;67(7):760-8.
