# Supplementary material for: A systematic review of neonatal treatment intensity scores and their potential application in low-resource setting hospitals for predicting mortality, morbidity and estimating resource use
Source: Syst Rev. 2017 Dec 7;6:248. doi: 10.1186/s13643-017-0649-6 (PMC5719732; doi:10.1186/s13643-017-0649-6)
Supplement: Supplementary file 4 — Using GRADE* as a guiding framework in rating of certainty of evidence in predictive model predictive performance. Description of the four categories in GRADE for rating certainty of evidence. *Grading of Recommendations Assessment, Development and Evaluation. (DOCX 13 kb) [file 13643_2017_649_MOESM4_ESM.docx]

**Addditional file 4: Table S3.** Using GRADE* as a guiding framework in rating of certainty of evidence in predictive model predictive performance

| Rating | Interpretation |
| --- | --- |
| 1. High certainty | Further research is very unlikely to change our confidence in the estimate of predictive performance |
| 1. Moderate certainty | Further research is likely to have an important impact on our confidence in the estimate of predictive performance and may change the estimate |
| 1. Low certainty | Further research is very likely to have an important impact on our confidence in the estimate of predictive performance and is likely to change the estimate |
| 1. Very low certainty | Any estimate of predictive performance is very uncertain |

* **G**rading of **R**ecommendations **A**ssessment, **D**evelopment and **E**valuation (**GRADE**)

Note: Adapted from Guyatt GH, Oxman AD, Vist GE, Kunz R et al. GRADE: an emerging consensus on rating quality of evidence and strength of recommendations. BMJ. 2008;336(7650):924-6.
