# Supplementary material for: A systematic review of neonatal treatment intensity scores and their potential application in low-resource setting hospitals for predicting mortality, morbidity and estimating resource use
Source: Syst Rev. 2017 Dec 7;6:248. doi: 10.1186/s13643-017-0649-6 (PMC5719732; doi:10.1186/s13643-017-0649-6)
Supplement: Supplementary file 5 — Summary of results from individual studies. Summary of data extracted from each eligible article. (DOCX 17 kb) [file 13643_2017_649_MOESM5_ESM.docx]

| Study | Predicting prognosis | Estimating resource utilization |
| --- | --- | --- |
| Georgieff et al,1989 | - Linear relationship between TISS* and PSI†; r=0.75, p<0.001 - TISS* decreased admission to discharge (<14-day stay;8.2±8.1 vs 3.1±2.9 and >14 day;16.9±8.1 vs 5.4±4.0, p<0.001) - Higher mean TISS* on day 1 and 5 in HMD^‡^ vs TTN^§^ (16.2 ± 9.4/11.9±7.1 vs 9.2 ± 6.0/3.8 ± 1.5) - Above median TISS* (≥7) longer to adequate nutritional intake (5.1 ± 3.3 days’ vs 1.9 ± 1.9 days, *p* < .001) - Days of inadequate nutritional intake correlated with first-day TISS* (r = .66, *p* < .001) | - Linear relationship TISS* and NUMIS^\|\|^; r=0.72, p<0.001 - Mean TISS scores increased with increase in NUMIS^\|\|^ classification (p<0.001) - Mean TISS* higher for level 3(NUMIS^\|\|^ 7-10) care vs level 2 (NUMIS^\|\|^ 5-6); 10.8±6.6 vs 3.2±1.4, p<0.001 |
| Gray et al,1992 | - NTISS associated with physician assessment of mortality (r=0.70, p<0.0001) - Increasing NTISS^¶^ categories associated with increased in-hospital mortality (χ2 for trend = 153.3, p<0.001) - All NTISS^¶^ sub-scores except transfusion therapy independently contributed to mortality (p <0.001) - Close agreement between observed and predicted mortality (H-L test) | - NTISS^¶^ (in survivors) correlated with - length of stay (all hospitals); r=0.37, p<0.0001 - Hospital charges (Hospital A); r=0.64, p<0.0001 - Nursing workload (Medicus**, Hospital A); r=0.69, p<0.0001 |
| Davies et al,1995 | - In-hospital mortality concurred with the predicted range for each risk group (A; predicted 5% actual 0, B; 20% vs 20%, C;30% vs 37%) | - Not reported |
| Eriksson et al,2002 | - NTISS^¶^ prediction of; in-hospital mortality; AUROC^††^ 0.82±0.04, early adverse outcome; AUROC^††^ 0.78±0.03, morbidity at 4 years; AUROC^††^ 0.59±0.05, overall adverse outcome; AUROC^††^ 0.70±0.04 | - NTISS^¶^ contact with rehabilitation; ROC Az 0.59±0.06 |
| Zupancic et al,2002 | - Not reported | - Nursing time inputs; predicted 42% of the variability (R^2^ = 0.42, p<0.0001) - Nursing & physicians combined; 23% of the variability (R^2^ = 0.23, p<0.0001) - Respiratory therapists; 36% of the variability (R^2^ = 0.36, p<0.0001) |
| Mendes et al ,2006 | - Not reported | - Mean daily value of overall NTISS^¶^; gradual and progressive downward trend up to the 31^st^ day, difference in curves after day 31 (increase in the public NICU; p<0.001) |
| Rojas et al 2011 | - Not reported | - Total NTISS^¶^ 5200 (SD: 70.10), mean 12.3 per measurement corresponding to 123 minutes of nursing interventions over a 6-hour period |
| Oygur ,2012 | - AUROC for predicting mortality with all variables; 0.851 (95% confidence interval [CI], 0.809–0.885); group 1,0.834 (95%CI:0.781–0.878); group 2 ,0.749 (95%CI: 0.662–0.822); group 3 - AUROC NTISS^¶^ only sensitive variables ;0.848 (95%CI: 0.806–0.883); group 1,0.821 (95%CI: 0.756–0.858); group 2 ,0.823 (95%CI: 0.744–0.886); group 3   - Increase in outcome significant only for group 3 patients for AUC NTISS^¶^ all variables vs NTISS^¶^ for only the sensitive variables (P = 0.02) | - - Not reported |
| Shah et al,2015 | - Crude and adjusted resource use on the day of admission associated with composite outcome - Odds of the composite outcome higher for infants admitted to an NICU^‡‡^ on day resource use in the highest quartile (1.28 95 C. I1.07-1.54), compared to day resource use in the lowest quartile (ref category 1) | - Not reported |
| Wu et al,2015 | - Average NTISS^¶^ scores at 24 hours, 48 hours, and 72hours higher in the mortality group (all p < 0.001) - Overall NTISS^¶^ scores at 24 hours, 48 hours, and 72 hours; 20.31± 5.6, 23.82± 6.2, 24.68± 5.8, (p < 0.001 one-way ANOVA). - Logistic regression NTISS^¶^ score at 48 hours’ difference between mortality and survivors (p =0.047) - AUC for NTISS^¶^ at 24 ,48 and 72 hours were 0.913, 0.955 and 0.958, respectively in predicting mortality - Optimal NTISS^¶^ score at 48 hours for predicting mortality was ≥28.0, sensitivity 100%, specificity 81.2% (p < 0.001) - Logistic regression NTISS^¶^ score at 48 hours AUROC 0.989, sensitivity of 100.0% and specificity of 96.1%. | - Not reported |

**Additional file 5: Table S4**. Summary of results from individual studies

* TISS: Therapeutic Intervention Scoring System, † PSI: Physiological Stability Index, ‡ HMD: Hyaline Membrane Disease, § TTN: Transient Tachypnoea of the Newborn, || NUMIS: Nursing Utilization Management Information System, ¶ NTISS: Neonatal Therapeutic Intervention Scoring System, **Medicus: nursing workload tool (Medicus System, Evanston) †† AUROC: Area Under the Receiver Operating Characteristic curve,

‡‡ NICU: Neonatal Intensive Care Unit
